# Blood Agar Validation for Susceptibility Testing of Isoniazid, Rifampicin, Ethambutol, and Streptomycin to *Mycobacterium tuberculosis* Isolates

**DOI:** 10.1371/journal.pone.0055370

**Published:** 2013-02-06

**Authors:** Ahmet Yilmaz Coban

**Affiliations:** Ondokuz Mayis University, Medical School, Department of Medical Microbiology, Samsun, Turkey; St. Petersburg Pasteur Institute, Russian Federation

## Abstract

In recent studies, it was shown that blood agar can be used at least as effectively as Löwenstein-Jensen medium for growing *Mycobacterium tuberculosis*. It was also shown that susceptibility testing can be performed on blood agar. Additional validation of blood agar was performed on regional *M. tuberculosis* isolates from Turkey to determine critical concentrations of isoniazid (INH), rifampicin (RIF), ethambutol (ETM), and streptomycin (STR). In the current study, 40 *M. tuberculosis* clinical isolates were tested. H37Rv, which is susceptible to all antituberculosis agents, ATCC 35822 (INH-resistant), ATCC 35838 (RIF-resistant), ATCC 35837 (ETM-resistant), and ATCC 35820 (STR-resistant) quality control strains were used as control strains. Proportion method on 7H11 agar was considered as gold standard in the study. MIC values of the control strains and clinical isolates were detected on blood and 7H11 agar. Categorical agreements were 100% for each antibiotic, and essential agreements were 100%, 97.5%, 82.5%, and 95% for INH, RIF, ETM, and STR, respectively. According to the data, 0.2 µg/mL for INH, 1 µg/mL for RIF, 4 µg/mL for ETM, and 2 µg/mL for STR were appropriate breakpoint values for susceptibility testing on blood agar. Blood agar may be recommended for use in both developed and developing countries for the susceptibility testing of *M. tuberculosis* isolates to primary antituberculosis drugs.

## Introduction

*Mycobacterium tuberculosis* is the oldest infectious agent known to humankind, and still remains a major public health issue [1]. Increased incidence of multidrug-resistant isolates has resulted in treatment failures and, these isolates were able to spread in the community. Recently, extensively drug-resistant strains have also been noted. In terms of public health, the most important step is the detection of the primary antituberculosis drug susceptibilities of the isolates early for the effective treatment of these isolates [2].

As of late, standard methods were adopted for defining the sensitivity of the primary antituberculosis agents. These methods are the proportion method performed on Middlebrook 7H10-11 agar and Löwenstein-Jensen media and commercial automated systems such as Bactec 460 TB and Bactec MGIT 960 [3], [4]. However, there are disadvantages to these methods. The Middlebrook 7H10 and 11 agar mediums used in these methods require growth-enhancing supplementation and incubation in carbon dioxide. Even, it requires at least 4 weeks incubation for reporting of susceptibility testing [4]. The preparation of Löwenstein-Jensen medium is difficult, and the content is likely to interact with antibiotics [5]. Rapid automated systems (Bactec 460 TB and BACTEC MGIT 960) cannot be obtained by laboratories with limited resources due to equipment requirements and high costs [6].

It was reported that mycobacteria were grown on blood agar in 1977 [7]. Nevertheless, it has not been considered for routine usage in clinical mycobacteriology laboratory. In recent years, it has been used for routine cultures in some laboratories [8]–[16]. Blood agar is inexpensive and can be easily supplied by many laboratories. Many bacteria can easily grow on blood agar and can be resulted in contamination during processing of clinical specimens (sputum etc.) for bacterial culture. In order to solve this problem, selective blood agar medium containing various antibiotics may be used.

The aim of this study is the further validation of blood agar for determining the critical concentrations of isoniazid (INH), rifampicin (RIF), ethambutol (ETM), and streptomycin (STM) for *M. tuberculosis* clinical isolates.

## Materials and Methods

In this study, the MIC values of the tested bacteria were determined by using agar dilution method on Middlebrook 7H11 agar and blood agar. Interpretation of MIC values was performed according to the Clinical and Laboratory Standards Institute criteria (document M24-A2) [4].

### Bacterial Isolates

Forty *M. tuberculosis* clinical isolates were tested. H37Rv, sensitive to all antituberculosis agents, ATCC 35822 (INH-resistant), ATCC 35838 (RIF-resistant), ATCC 35837 (ETM-resistant), and ATCC 35820 (STR-resistant) strains were tested for quality control. Fresh cultures on Löwenstein-Jensen medium (2–3 weeks) of all isolates were used.

### Antibiotics and Preparation

INH, RIF, STR, and ETM were purchased from Sigma (Sigma-Aldrich, Germany). All antibiotics stocks were prepared to be 16.384 µg/mL. All antibiotics except RIF were dissolved in sterile distilled water, sterilized by filtration, and stored at −80°C until use. RIF was dissolved in methanol and then stored at −80°C until use.

### Preparation of Bacterial Inoculums

Colonies from fresh Lowenstein-Jensen culture were transferred into screw cap tubes containing 5–10 glass beads and 5 mL of saline solution. Tubes were vortexed for 30 seconds. Tubes were kept at room temperature for 30 minutes in an upright position. Thus, large particles and aerosols were collapsed. Then, the supernatants were transferred to another tube and adjusted to McFarland no. 1 turbidity and were diluted 1∶100. The final dilution was used as the inoculums source for both mediums.

### Preparation of Blood and Middlebrook 7H11 Agars

Middlebrook 7H11 medium (BD, Becton Dickinson and Company, Sparks, MD, USA) was prepared according to manufacturer instructions, sterilized by autoclaving, and cooled to 50°C. Oleic acid, albumin, dextrose, catalase (OADC) were added into medium at 10%. Then, the antibiotics were added to the medium. Blood agar medium (bioMerieux, Marcy I’Etoile, France) was prepared according to manufacturer recommendations, glycerol was added up to 5 mL/liter, and then sterilized by autoclaving and cooled to 50°C. Defibrinated sheep blood was purchased from Pendik Veteriner Kontrol Enstitusu, Pendik, Istanbul, Turkey, and it (5%) was added to the medium. Then, the antibiotics were added to medium. All isolates were tested at 0.03–32 µg/mL concentrations for all antibiotics. After adding the desired antibiotic concentration, 1 mL of media were dispersed into 24-well plates and solidified. Antibiotic-free control media were prepared for each bacterium. Plates were stored at 4°C until use. The storage period did not exceed 4 weeks.

### Testing of Isolates

Prepared inoculum (20 µL) of bacteria was added to each well. After inoculation, plates of 7H11 agar were incubated at 37°C under condition of 5% CO_2_; plates of blood agar were incubated at 37°C under normal atmospheric air. After the second day of incubation they were observed for growth (including contamination). Then, the plates were observed every 3 days. On the 21st day of incubation, if there were more than 50 colonies in the control well, the results were noted. MIC was defined as the lowest concentration of the drug that inhibited the bacterial growth in the well. In addition, the categorical results for each isolate according to the critical concentration recommended by CLSI were recorded as sensitive or resistant. Categorical results were calculated by comparing the colony number of the drug-containing tube with the colony number of the drug-free tube. If this ratio was >1%, INH, RIF, ETM and STR were considered resistant [3], [4], [17]. Thus, MIC values and categorical resistance/susceptibility patterns were reported for each strain.

### Data Analysis

Data were evaluated according to FDA criteria [18]. *Essential agreement (EA);* agreement within plus or minus, one two-fold dilution of the new device under evaluation with the reference method MIC determination. *Categorical agreement (CA);* agreement of interpretive results (SIR) between a new device under evaluation and a standard reference method using FDA interpretive criteria as presented in the FDA approved pharmaceutical antimicrobial agent package insert. *Major discrepancy (maj);* the reference category result is S and the new device result is R. *Minor discrepancy (min);* the reference category result is R or S and the new device result is I; or the reference result is I and the new device result is R or S. *Very major discrepancy (vmj)*; the reference category result is R and the new device result is S. Categorical agreement (CA), essential agreement (EA), minor discrepancy (min), major discrepancy (maj), and very major discrepancy (vmj) were calculated.

In addition, specificity, sensitivity, positive predictive values (PPV) and negative predictive values (NPV) were also calculated.

## Results

In the study, 40 *M. tuberculosis* clinical isolates were tested. The MIC values of tested standard strains determined in both media are summarized in Table 1.

**Table 1:** MIC values of standard strains on both media in the preliminary study.

Strains	INH (µg/ml)		RIF (µg/ml)		ETM (µg/ml)		STR (µg/ml)	
	BA	7H11A	BA	7H11A	BA	7H11A	BA	7H11A
H37Rv	0.125	0.125	1	1	4	4	2	1
ATCC 35822	>32	>32	1	1	4	4	2	1
ATCC 35838	0.125	0.125	>32	>32	4	4	2	1
ATCC 35820	0.125	0.125	1	1	4	4	>32	>32
ATCC 35837	0.06	0.06	0.5	0.25	16	16	1	1

INH: isoniazid; RIF: rifampicin; ETM: ethambutol; STR: streptomycin; BA: Blood agar; 7H11A: 7H11 agar; S: susceptible;

R: resistant.

The MICs of 40 clinical isolates obtained from blood agar were compared with the MICs obtained from 7H11 agar that was used as gold standard (Table 2). INH MIC values were the same in 22 isolates, while they were within ±1 dilution in the remaining 18 isolates. Both categorical and essential agreements were 100%. The MIC values of RIF were the same in 31 isolates, were within ±1 dilution in 8 isolates, and were within ±2 dilutions in 1 isolate. Categorical agreement and essential agreement were 100% and 97.5%, respectively. The MIC values of ETM were the same in 18 isolates; within ±1 dilution in 15 isolates; within ±2 dilutions in 5 isolates; and within ±3 dilutions in 2 isolates. Categorical agreement and essential agreement were 100% and 82.5%, respectively. The STR MIC values were same in 22 isolates; within ±1 dilution in 16 isolates; and within ±2 dilutions in 2 isolates. Categorical agreement and essential agreement were 100% and 95%, respectively. There was no very major, major, or minor discrepancy (Table 2). For all antibiotics, specificity, sensitivity, PPV, and NPV values were 100%.

**Table 2:** Comparison of the MIC values of each antibiotics on blood agar with 7H11 agar.

Drugs	0 dilution (n)	±1 dilution (n)	±2 dilution (n)	±3 dilution (n)	EA (%)	CA (%)
INH	22	18	–	–	100	100
RIF	31	8	1	–	97.5	100
ETM	18	15	5	2	82.5	100
STR	22	16	2	–	95	100

EA: essential agreement, CA: Categorical agreement; n: number of isolates.

MIC distribution of the isolates for each antibiotic on both media was compared in Table 3. Fourteen out of 40 isolates were resistant to INH and, resistance was detected in all on both blood and 7H11 agar. Critical concentration of INH as recommended by the CLSI is 0.2 µg/mL. The MIC values of 14 INH-resistant isolates tested in this study were ≥0.5 µg/mL on both media (Table 3). In conclusion, the critical concentration of INH was detected as 0.2 µg/mL for blood agar.

**Table 3:** Distribution of drugs MICs on both media.

Concentrations of antibiotic (µg/ml)														Total
		0.03 (n)	0.06 (n)	0.125 (n)	0.25 (n)	0.5 (n)	1 (n)	2 (n)	4 (n)	8 (n)	16 (n)	32 (n)	>32 (n)	(susceptible/resistant) (n)
BA	INH	0	15(S)	10(S)	1(S)	2(R)	2(R)	1(R)	3(R)	4(R)	2(R)	0	0	26/14
	RIF	0	0	0	2	5	18	1(S)/2(R)	0	1(R)	0	1(R)	10(R)	26(S)/14(R)
	ETM	0	0	0	0	0	1(S)	7(S)	20(S)	3(S)	2(S)/5(R)	1(R)	1(R)	33(S)/7(R)
	STR	0	0	0	2(S)	8(S)	15(S)	5(S)	1(S)	6(R)	2(R)	0	1(R)	31(S)/9(R)
MB	INH	0	9(S)	13(S)	4(S)	2(R)	2(R)	0	1(R)	6(R)	3(R)	0	0	26/14
	RIF	0	0	0	1	7	14	3(S)	1(S)/2(R)	1(R)	0	1(R)	10(R)	26(S)/14(R)
	ETM	0	0	0	0	0	0	2(S)	14(S)	10(S)	4(S)/5(R)	3(S)/1(R)	1(R)	33(S)/7(R)
	STR	0	0	0	0	13(S)	16(S)	2(S)	3(R)	3(R)	2(R)	0	1(R)	31(S)/9(R)

BA: Blood agar; MB: Middlebrook 7H11 agar; S: susceptible; R: resistant (R and S were defined according to proportion method); n: number of isolates.

RIF resistance was detected in 14 out of 40 isolates on both blood and 7H11 agar. The critical concentration of RIF as recommended by the CLSI is 1 µg/mL. The MIC values of 14 RIF-resistant isolates tested in this study were ≥1 µg/mL on both media. However, the MIC value of a RIF-sensitive isolate was 2 µg/mL on blood agar but 4 µg/mL on 7H11 agar. This isolate was susceptible on both media according to CLSI criteria (Table 3). It was also found susceptible by automated Bactec MGIT 960 (Becton Dickinson, Sparks, MD, USA). The MIC values of 3 isolates were 2 µg/mL on 7H11 agar whereas they were 1 µg/mL on blood agar. These isolates were susceptible according to CLSI criteria and Bactec MGIT 960 (Becton Dickinson, Sparks, MD, USA). In conclusion, the critical concentration of RIF was detected as 1 µg/mL for blood agar.

In 7 ETM resistant isolates, resistance was detected on both blood and 7H11 agar. The critical concentration of ETM as recommended by the CLSI is 7.5 µg/mL. The MIC values of 7 ETM-resistant isolates tested in this study were ≥16 µg/mL on both media. The MIC value of ETM was 16 µg/ml in 2 isolates on blood agar whereas it was 16 µg/mL in 1 and 32 µg/mL in remaining isolate on 7H11 agar. However, the isolates were susceptible on both media according to CLSI criteria. The MIC value of 2 out of 5 sensitive isolates was 32 µg/mL, and the MIC value of the remaining 3 isolates was 16 µg/mL on 7H11 agar. The MIC value of these 5 isolates was 4 µg/mL on blood agar (Table 3). There were 4–5 colonies in wells containing 16 and 32 µg/mL concentrations of ETM in which isolates showed high MIC value on 7H11 agar. The ETM MIC values on blood agar were lower than in the 2–3 dilution on 7H11 agar. In conclusion, the critical concentration of ETM was detected as 4 µg/mL for blood agar.

In 9 STR resistant isolates, resistance was detected on both blood and 7H11 agar. The recommended critical concentration of STR by the CLSI is 2 µg/mL. The MIC values of 9 STR-resistant isolates tested in this study were ≥4 µg/mL on both media. Although the MIC value of a sensitive isolate was 4 µg/mL on blood agar, it was susceptible to STR according to CLSI criteria. The MIC value of this isolate was 1 µg/mL on 7H11 (Table 3). When this isolate was tested by Bactec MGIT 960 (Becton Dickinson, Sparks, MD, USA), it was found susceptible to STR. In conclusion, the critical concentration of STR was detected as 2 µg/mL for blood agar.

MIC values of some strains were higher than critical concentrations however they were classified as susceptible according to CLSI criteria. There were 1–5 bacterial colonies in wells contained critical concentrations, whereas there were +4 (>500 confluent colonies) in control wells for tested all isolates. Therefore resistance was below 1%.

## Discussion

Tuberculosis is a disease that is more common in developing countries. Early detection of resistance patterns of isolated tubercle bacilli and implementation of an effective treatment protocol are the most critical steps to preventing the spread of disease in these populations. Therefore, there is needed the methods for the determination of resistance that are easily prepared and applied and that utilize inexpensive media. Because blood agar has these features, it can be prepared easily and cheaply, it can be used successfully in many laboratories [10].

In the mycobacteriology laboratories of many developing countries egg-based Löwenstein-Jensen medium is used as a solid medium for determining antituberculosis drug susceptibility in clinical isolates of *M. tuberculosis*. In addition, laboratories which have more advanced facilities use broth-based automated systems. In recent studies, it has been shown that blood agar, a basic medium in clinical microbiology laboratories, can also be used for growth of mycobacteria [13]–[16].

Drancourt et al. [14] reported that *M. tuberculosis* isolates were easily grown in 1–2 weeks on blood agar, and the number of colonies obtained from clinical samples on blood agar was greater than in egg-based medium. Drancourt and Raoult [15] compared the Bactec 9000 MB system with blood agar in terms of time and price. While the cost of blood agar was €1913 for the analysis of 1000 samples, it was increased to € 8990 with the Bactec 9000 MB system. Time of detection was 19±5 days for blood agar and 22±6 days for the Bactec 9000 MB system. In their study, growth of 41 different mycobacterial species was evaluated on blood and Middlebrook agar. It was reported that *M. ulcerans* was not grown on blood agar, whilst *M. haemophilum* was not grown on Middlebrook agar. In the study, it was shown that mycobacteria species can be easily grown on blood agar. Species among mycobacteria cannot be differentiated according to colony morphologies on blood agar.

After it was shown that tubercle bacilli grow on blood agar, antibiotic susceptibility studies were performed on blood agar. Coban et al. [8] performed susceptibility testing of INH and RIF on blood agar; agreements were 100% and 94.1% for INH and RIF, respectively. Coban et al. [9] investigated the susceptibility of *M. tuberculosis* isolates to INH, RIF, ETM, and STR on blood agar and compared it with the standard method. The agreements for INH, RIF, ETM, and STR were 100%, 100%, 96%, and 92%, respectively.

Yildiz et al. [19] showed that both sheep blood and human blood agar can be used for determination of INH sensitivity. Coban et al. [12] compared susceptibility results on blood agar with the results from the standard method in a multicenter study. The agreement for INH, RIF, ETM, and STR were 94.5%, 96.3%, 97.1%, and 93.3%, respectively.

The susceptibility of *M. tuberculosis* isolates to first and second line antituberculosis drugs was investigated with Middlebrook 7H11 agar, blood agar, and chocolate agar by E-test. It was reported that blood agar can be used just as Middlebrook 7H11 agar [10]. Satana et al. [20] used blood agar for susceptibility testing of *M. tuberculosis* clinical isolates to second line antituberculosis drugs including kanamycin, rifabutin, ofloxacin, p-aminosalicylic acid, capreomycin, clofazimine, and ethionamide. At the end of the study agreement with the standard method was 86%–100%.

In light of the above information and the current study, additional validation of blood agar was performed on regional *M. tuberculosis* isolates from Turkey to determine critical concentrations of INH, RIF, ETM, and STR. Categorical results of the isolates tested in this study were performed according to CLSI criteria [4].

In a number of bacteria the susceptibility results are reported as follows: if the MIC is greater than the breakpoint it is resistant, and if it is less than the breakpoint it is susceptible. However, such criterion is not used in determining the susceptibility of *M. tuberculosis*; instead, a single critical concentration is used [4]. When evaluating test results, it is not necessarily the absence of any growth in the concentration that should be evaluated primarily. Instead, if there is growth in the critical concentration (breakpoint) should be assessed to be compared with the colony number in the control (1% for INH, RIF, ETM and STR). Accordingly, the results are reported as sensitive or resistant [4]. Therefore, although the MIC values of ETM were 16–32 µg/mL in 7 isolates, they were susceptible according to CLSI criteria. There were 3–5 colonies in wells containing 16 and 32 µg/mL concentrations of ETM. In addition, when these isolates were tested by Bactec MGIT 960 (Becton Dickinson, Sparks, Md., USA) they were still sensitive to ETM.

EMB susceptibility testing has been historically more difficult to reproduce given MICs near the critical concentration in many strains. It was reported that most strains that had microcolonies with EMB in the agar proportion method had EMB-susceptible results with BACTEC 460TB. The significance of microcolonies is unknown [4]. In the comparative study, EMB resistant isolates have been discriminated from susceptible isolates at 2 µg/ml in the laboratory of the Korean Institute of Tuberculosis, while they were discriminated at 4 µg/ml in the laboratory of the Royal Postgraduate Medical School in the same isolates, using the same medium and method [21]. In this study, resistant isolates were better distinguished at 4 µg/ml.

In light of the data, the critical concentrations for blood agar are easily applicable for susceptibility testing for clinical isolates of *M. tuberculosis*. Therefore, blood agar can be recommended for determining the sensitivity of the primary antituberculosis agents in both developed and developing countries.

## References

[1] Harries AD, Dye C (2006) Tuberculosis. Ann Trop Med Parasitol 100: 415–431. 16899146 10.1179/136485906X91477

[2] Palomino JC, Leao SC, Ritacco V (2007) Tuberculosis 2007. Chapter 19: Drug resistance and drug resistance detection, p635–655.

[3] Canetti G, Fox W, Khomenko A, Mahler HT, Menon NK, et al (1969) Advances in techniques of testing Mycobacterial drug sensitivity, and the use of sensitivity tests in tuberculosis control programmes. Bull WHO 41: 21–43. 5309084 PMC2427409

[4] Clinical and Laboratory Standards Institute (CLSI) (2011) Susceptibility testing of Mycobacteria, Nocardiae, and other aerobic Actinomycetes; Approved standard- Second edition. M24-A2, Wayne, Pennsylvania USA. 31339680

[5] Kent PT, Kubica GP (1985) Public Health Mycobacteriology. A Guide for Level III Laboratory. Atlanta, GA: CDC, p47.

[6] Coban AY, Tanriverdi Cayci Y, Deveci A, Akgunes A, Uzun M, et al (2011) A rapid detection of multidrug-resistant *Mycobacterium tuberculosis* by a nitrate reductase assay on blood agar. Mem Ins Oswaldo Cruz 106: 378–380. 10.1590/s0074-02762011000300022 21655831

[7] Kilicturgay K, Gumrukcu E, Tubluk F, Saglam M (1977) The results in our tuberculosis laboratory with penicillin blood agar medium. Mikrobiyol Bul 11: 29–33. 404514

[8] Coban AY, Bilgin K, Uzun M, Tasdelen Fisgin N, Akgunes A, et al (2005) Susceptibilities of *Mycobacterium tuberculosis* to isoniazid and rifampin on blood agar. J Clin Microbiol 43: 1930–1931. 15815022 10.1128/JCM.43.4.1930-1931.2005 PMC1081341

[9] Coban AY, Cihan CC, Bilgin K, Uzun M, Akgunes A, et al (2006) Blood agar for susceptibility testing of *Mycobacterium tuberculosis* against first-line drugs. Int J Tuberc Lung Dis 10: 450–453. 16602412

[10] Coban AY, Bilgin K, Uzun M, Akgunes A, Yusof A, et al (2008) Comparative study for determination of *Mycobacterium tuberculosis* susceptibility to first- and second-line antituberculosis drugs by the Etest using 7H11, blood, and chocolate agar. J Clin Microbiol 46: 4095–4098. 18945843 10.1128/JCM.01104-08 PMC2593295

[11] Coban AY, Bilgin K, Akgunes A, Durupinar B (2008) Susceptibility testing of *Mycobacterium tuberculosis* isolates to primary antituberculous drugs on chocolate agar: a preliminary study. Mikrobiyol Bul 42: 477–481. 18822892

[12] Coban AY, Martin A, Uzun M, Bilgin K, Palomino JC, et al (2008) Evaluation of blood agar for susceptibility testing of *Mycobacterium tuberculosis* against first-line antituberculous drugs: results from two centers. J Chemother 20: 388–390. 18606599 10.1179/joc.2008.20.3.388

[13] Coban AY, Akgunes A, Durupinar B (2011) Evaluation of blood agar medium for the growth of Mycobacteria. Mikrobiyol Bul 45: 617–622. 22090292

[14] Drancourt M, Carrieri P, Gévaudan MJ, Raoult D (2003) Blood agar and Mycobacterium tuberculosis: the end of a dogma. J Clin Microbiol 41: 1710–1711. 12682165 10.1128/JCM.41.4.1710-1711.2003 PMC153881

[15] Drancourt M, Raoult D (2007) Cost-effectiveness of blood agar for isolation of mycobacteria. PLoS Negl Trop Dis 28 1: e83 http://www.ncbi.nlm.nih.gov/pubmed/18060087. 10.1371/journal.pntd.0000083 PMC2100370 18060087

[16] Mathur ML, Gaur J, Sharma R, Solanki A (2009) Rapid culture of *Mycobacterium tuberculosis* on blood agar in resource limited setting. Dan Med Bull 56: 208–210. 19939338

[17] Barreto AMW, Araujo JBM, Medeiros RFM, Caldas PCS (2003) Evaluation of indirect susceptibility to the first- and second-line, and alternative drugs by the newer MB/BacT system. Mem Inst Oswaldo Cruz 98: 827–830. 14595463 10.1590/s0074-02762003000600020

[18] U.S. Food and Drug Administration (FDA) website. Available: http://www.fda.gov/MedicalDevices/DeviceRegulationandGuidance/GuidanceDocuments/ucm080564.htm. Accessed 2013 Jan 7.

[19] Yildiz C, Ulger M, Aslan G, Emekdas G (2007) Isoniazid susceptibilities of Mycobacterium tuberculosis on blood agar. Saudi Med J 28: 975–977. 17530127

[20] Satana D, Coban AY, Uzun M (2010) Testing susceptibility of multidrug-resistant *Mycobacterium tuberculosis* to second-line drugs by use of blood agar. J Clin Microbiol 48: 4291–4293. 20826642 10.1128/JCM.00688-10 PMC3020831

[21] Kim SJ (2005) Drug-susceptibility testing in tuberculosis: methods and reliability of results. Eur Respir J 25: 564–569. 15738303 10.1183/09031936.05.00111304

